# Spatiotemporal Emission Characteristics and Driving Factors of Air Pollutants and CO_2_ from Aircraft at Airports: A Case Study in China

**DOI:** 10.3390/toxics14090751

**Published:** 2026-08-26

**Authors:** Yapeng Li, Mingwei Zhang, Yinxiao Zhang, Zhongcun Han, Jian Chen, Yuzhe Liu, Runjie Liu, Jiahui Xu, Yueyuan Niu, Wei Hao, Wenlai Ma, Weijun Li

**Affiliations:** 1 Flight College, Shandong University of Aeronautics, Binzhou 256600, China; 2 Shandong Key Laboratory of Eco-Environmental Science for the Yellow River Delta, Shandong University of Aeronautics, Binzhou 256600, China; 3 CAST Zhongyu (Beijing) New Technology Development Co., Ltd., Beijing 101200, China; 4 Department of Atmospheric Sciences, School of Earth Sciences, Zhejiang University, Hangzhou 310027, China

**Keywords:** aviation emissions, civil aviation airports, emission inventory, LTO cycle, driving factors, Shandong Province

## Abstract

With the rapid expansion of China’s civil aviation industry, airport aircraft emissions have raised growing environmental concerns, making it imperative to clarify their emission characteristics and driving mechanisms. In this study, 10 major civil aviation airports in Shandong Province, a highly representative airport cluster in China, were selected to investigate the characteristics of aviation emissions using actual flight activity data from 2023. Our results show that the total emissions of HC, CO, NO_x_, SO_2_, PM, and CO_2_ from the airport aircraft were 144.6, 1890.4, 2571.8, 358.7, 18.2, and 584,822 tons, respectively, with NO_x_ and CO being the dominant air pollutants. The taxiing phase was identified as the largest contributor to total emissions, and the B737-800 emerged as the largest emission-contributing aircraft type. Moreover, our results reveal that airport aircraft emissions exhibited spatiotemporal heterogeneity. These emissions peaked in July and August, but displayed low levels during wintertime. Spatially, these aviation emissions were predominantly concentrated in eastern Shandong Province, and Qingdao and Jinan cities were the top two contributors, jointly contributing more than 66% of the total emissions. This study further indicates that this spatiotemporal heterogeneity was primarily attributed to air passenger throughput, aircraft movements, and GDP. Our findings can be extended to other provinces in China and provide a scientific basis for the future mitigation of aviation emissions at airports across China.

## 1. Introduction

With the rapid development of economic globalization, aviation transportation has become a key component of the modern integrated transportation system, triggering remarkable global industry expansion. According to the report of the International Air Transport Association (IATA), global air passenger traffic turnover has increased from 17 billion in 2010 to 45 billion in 2019, and it will continue to grow at an annual rate of 4.3% [1,2]. Meanwhile, China’s aviation sector has also undergone rapid expansion, with the number of transport airports increasing from 135 to 263 and passenger throughput rising from 0.28 billion to 1.46 billion between 2005 and 2024 [3]. Although affected by the COVID-19 pandemic during 2020 and 2022, the average annual passenger traffic turnover still reached 0.37 billion [4,5]. Driven by the global economic recovery, global demand for air transport services is projected to increase substantially. According to the report of Airbus, the global aviation market is expected to grow at 3.6% annually over the next two decades, and will require 40,850 new passenger and cargo aircraft [6]. Notably, China will account for nearly a quarter of this global demand and will surpass the United States to become the world’s largest aviation market by 2042 [6].

Some studies showed that this rapid development of civil aviation was bound to result in severe atmospheric environmental pollution problems [5,7,8]. A primary problem is the release of different types of toxic air pollutants, including hydrocarbons (HC), carbon monoxide (CO), nitrogen oxides (NO_x_), particulate matter (PM), and sulfur dioxide (SO_2_) in airports. Moreover, carbon dioxide (CO_2_) emissions from aviation have also become a critical global concern due to their significant contribution to climate change, and its emission has accounted for approximately 2.8% of global carbon emissions, with this share projected to rise substantially in the absence of effective mitigation measures [9,10,11]. A recent study has revealed that China’s aviation industry emitted a large amount of air pollutants, and approximately 19,000 tons of HC, 753,000 tons of NO_x_, 2400 tons of PM, 30,600 tons of SO_2_, 1000 tons of black carbon (BC), and 117 million tons of CO_2_ were emitted from aviation in China in 2018, accounting for nearly 20% of global aviation pollutant emissions [11].

These high levels of aviation emissions could significantly degrade air quality and pose adverse effects on human health, especially at airports and their surrounding areas [12,13,14]. It has been reported that aircraft operations could elevate ambient particle concentration by 2–10 times at airports [14] and even at 660 m downwind of the airport, ultrafine particle concentrations still exceeded the background values by 2.5 times [15]. These higher pollution levels posed more serious health risks to residents near airports, and it has been estimated that approximately one-third of aviation-related premature deaths were attributed to PM_2.5_ exposure within 20 km of the airports [16]. To improve the air quality at airports and protect the health of nearby residents, it is crucial to investigate the emission characteristics of air pollutants from aircraft at airports and then identify their driving factors.

In recent years, several studies have focused on aircraft air pollutant emissions at airports [8,17,18,19]. For example, Han et al. [20] investigated the aircraft emissions at Zhengzhou Xinzheng International Airport and revealed that during the LTO phase, these aircraft emissions of HC, CO, NO_x_, SO_2_, and PM were 56.3, 577.0, 969.4, 268.3, and 26.9 tons, respectively, contributing approximately 10% to the local NO_x_ concentrations. Moradi et al. [21] applied the AMS/EPA regulatory model (AERMOD) dispersion model to assess aircraft emissions from Imam Khomeini International Airport, and found that westerly and northwesterly winds exacerbated the impact of emissions on nearby residential and industrial areas. Lin et al. [22] combined a high-resolution emission inventory with the Weather Research and Forecasting model coupled to the Community Multiscale Air Quality (WRF-CMAQ) to evaluate aircraft emissions of Shenzhen Bao’an International Airport. However, these studies only focused on individual airports. Currently, studies on emissions from regional airport clusters are still scarce. Understanding the emission differences in multiple airports within a large region and their underlying driving factors is a key factor for the atmospheric pollution control of regional airports.

To address these gaps, this study investigated the emission characteristics of air pollutants and CO_2_ from 10 civil aviation airports in Shandong Province based on real flight data and the International Civil Aviation Organization (ICAO) standard emissions model. Then, we analyzed the spatiotemporal heterogeneity of these emissions in 10 airports. Finally, we employed the Multiscale Geographically Weighted Regression (MGWR) model to explore the key driving factors of aircraft emission differences in multiple airports in Shandong Province.

## 2. Methodology

### 2.1. Information About Airports and Flight Activity Data

**Figure 1:**
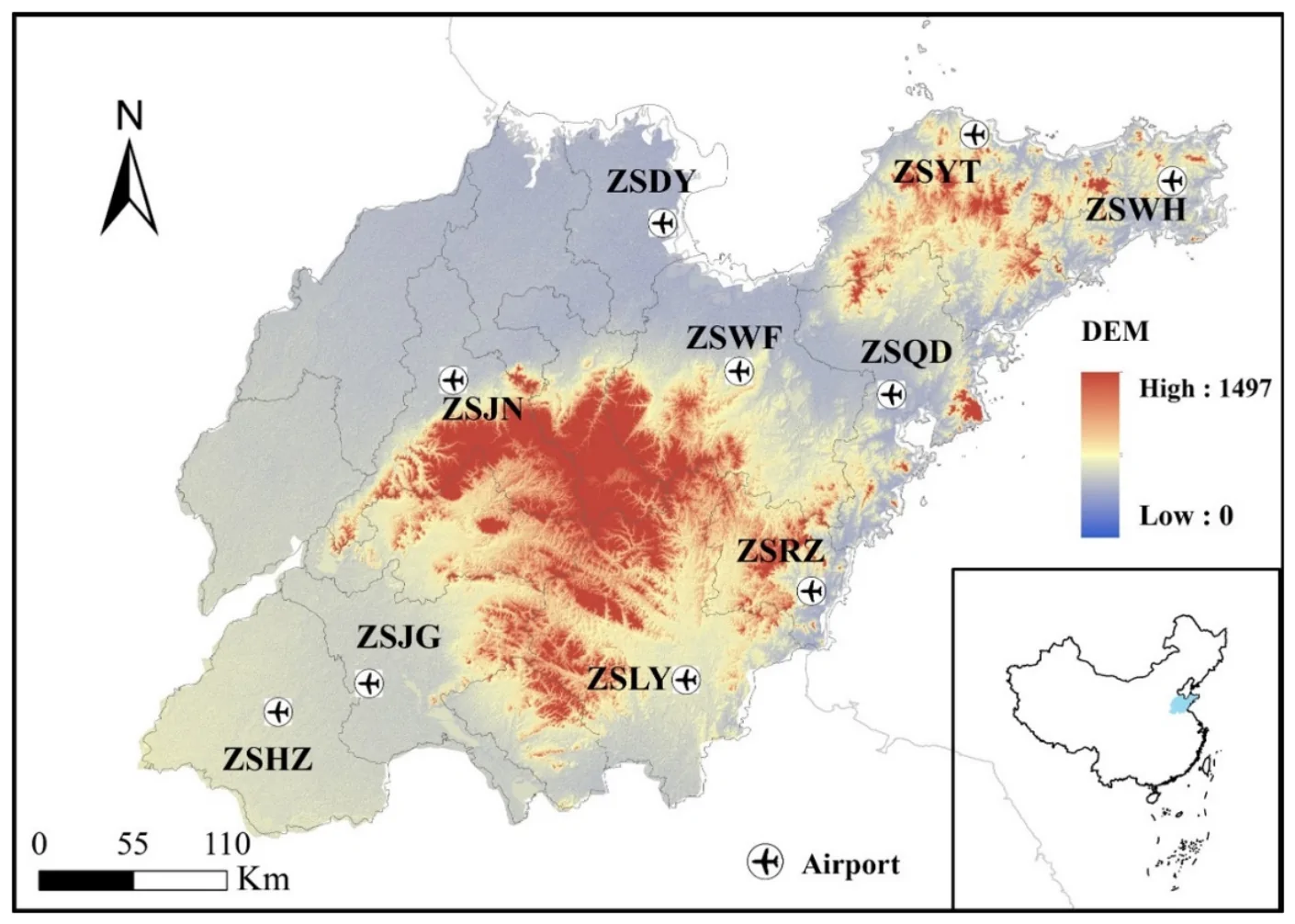
The spatial distribution of 10 major civil aviation airports in Shandong Province.

In this study, an airport cluster in Shandong Province was selected as a case to investigate the emission characteristics of airport aircraft air pollutants. Shandong Province is located in the eastern part of China (Figure 1), and it is one of the most economically developed and fastest-growing provinces in China. As an economic development hub, the civil aviation industry in Shandong Province is relatively developed. According to statistical data, there are 10 civil aviation transport airports and 22 general aviation airports in Shandong Province, making it one of China’s regions with the highest airport density [23]. Moreover, a report shows that the passenger throughput of 10 civil aviation airports in Shandong Province reached 55.83 million passengers, the cargo and mail throughput was 53.38 million tons, and the annual flight departures and landings reached 649,300 flights [24]. Therefore, the airport cluster in Shandong Province is highly representative in China.

Ten major civil aviation airports in Shandong Province were selected in this study, including Qingdao Jiaodong International airport (36°21′55″ N, 120°05′43″ E; IATA: TAO; ICAO: ZSQD; 4F), Jinan Yaoqiang International airport (36°51′07′′ N, 117°12′49′′ E; IATA: TNA; ICAO: ZSJN; 4E), Yantai Penglai International Airport (37°39′36″ N, 120°59′28″ E; IATA: ZSYT; ICAO: YNT; 4E), Weihai Dashuipo International Airport (37°11′20″ N, 122°14′22″ E; IATA: ZSWH; ICAO: WEH; 4D), Linyi Qiyang International Airport (35°02′55″ N, 118°24′55″ E; IATA: ZSLY; ICAO: LYI; 4D), Weifang Nanyuan Airport (35°24′06″ N, 119°19′45″ E; IATA: ZSWF; ICAO: WEF; 4D), Dongying Shengli Airport (36°38′31″ N, 119°07′12″ E; IATA: ZSDY; ICAO: DOY; 4D), Jining Da’an Airport (35°17′52″ N, 116°21′23″ E; IATA: ZSJG; ICAO: JNG; 4C), Rizhao Shanzihe Airport (37°30′21″ N, 118°47′15″ E; IATA: ZSRZ; ICAO: RIZ; 4C), and Heze Mudan Airport (35°12′50″ N, 115°44′17″ E; IATA: ZSHZ; ICAO: HZA; 4C). More detailed information about these airports has been presented in Figure 1 and Table S1.

In this study, we obtained a total of 431,967 pieces of actual flight activity data of these airports in 2023 from the China Civil Aviation Flight Operational Quality Assurance (FOQA) Monitoring System. This system can provide real-time flight data daily from over 4000 commercial aircraft operated by all civil transport airlines in China, thereby enabling comprehensive operational monitoring of nationwide aviation activities. The aircraft types and their engine types are the key factors affecting aircraft emissions. Figures S1 and S2 have further illustrated the fleet composition and engine types in Shandong airports. We found that the mainstream aircraft types in Shandong airports were B737 and A320 (Figure S1), accounting for 48.7% and 36.7% of total flight volume, respectively, and other aircraft types were less than 10%, including A321, A319, ERJ190 and ARJ21 (Figure S2). In more detail, the B737-800 was the most frequently used aircraft type, comprising 44.5% of all flights (Figure S2). For engine types, we found that the most widely used engine type was the CFM56 series engines, accounting for 65% of the total engine types. Other engine types were less than 15%, including V2500 (12.9%), LEAP-1A (10.7%), PW1127G (3.2%), and CF34-10 (2.3%) (Figure S2). It should be noted that CFM56-7B accounted for 46.1% of all engines, indicating its predominant position (Figure S2).

### 2.2. Estimation of Aircraft Emissions

Aircraft emissions in airports mainly refer to the emissions during the aircraft’s Landing and Take-Off (LTO) phase [22,25]. According to the International Civil Aviation Organization (ICAO) standard emission model, the LTO phase is categorized into four operational subphases: take-off, climb-out, approach, and taxiing. This phase-specific segmentation aligns with globally recognized methodologies for aviation emission assessments, ensuring consistency in quantifying pollutant emissions during critical low-altitude operations [26,27,28].

In this study, we adopted emission factors (*EF*) and the number of LTO phases to estimate emissions of multiple pollutants and CO_2_, referring to Equations (1) and (2). Additionally, Microsoft Excel (Microsoft Corporation, Redmond, WA, USA) was used for emission inventory calculations, while Origin 2024 (OriginLab, Northampton, MA, USA) and ArcMap 10.8.2 (Esri, Redlands, CA, USA) were used for data visualization.

(1) $${EF}_{i,j,k}={EI}_{i,j,k}\times {FF}_{k}\times \left({TIM}_{k}\times 60\right)\times n$$

(2) $$E_{i}=\sum_{k}\begin{array}{c} {EF}_{i,j,k} \end{array}\times M$$

where *EI*, *FF*, and *TIM* refer to emission index (g/kg fuel), fuel flow (kg/s), and time-in-mode (min), respectively; the subscripts i, j and k represent the pollutant types, aircraft engine types, and subphases of the LTO phase (take-off, climb-out, approach, taxiing), respectively; *EF* is the emission factor of pollutants in units of g; n and M are the numbers of engines and LTO phase, respectively. In this study, *FF* values were acquired from the ICAO Engine Emission Databank (EEDB), and *TIM* values for each subphase of the LTO phase were adopted from ICAO standards (Table S2). For HC, CO, and NO_x_, their *EIs* refer to the ICAO EEDB [29]. The *EIs* of CO_2_ and SO_2_ were adopted as 3155 g/kg fuel and 3.86 g/kg fuel, respectively [30,31].

Due to the absence of particulate matter (PM) emission indices in the EEDB, this study employed the first-order approximation method (FOA3.0) to estimate the emission indices of both volatile particulate matter and non-volatile particulate matter by using the smoke number and air-fuel ratio of the engine [32,33]. The ${EI}_{PM}$ was first calculated according to Equations (3)–(6). Then, the total PM emissions were acquired using Equations (1) and (2).

(3) $${EI}_{vol-O}=\delta \times {EI}_{HC}\times 10^{-3}$$

(4) $${EI}_{vol-S}=FSC\times \varepsilon \times 3\times 10^{3}$$

(5) $${EI}_{nvol}=0.694\times 10^{-4}\times {SN}^{1.234}\times \left(0.776\times AFR+0.877\right)$$

(6) $${EI}_{PM}={EI}_{vol-O}+{EI}_{vol-S}+{EI}_{nvol}$$

Here, ${EI}_{vol-O}$ represents the EIs of the organic component in non-volatile particulate matter (g·kg^−1^). ${EI}_{vol-s}$ represents the EIs of the sulfur component in volatile particulate matter (g·kg^−1^). ${EI}_{nvol}$ is the EI of non-volatile particulate matter components (g·kg^−1^). δ is the ratio of specific phase during the LTO phase, using the default values (Table S2). *FSC* refers to the sulfur content in aviation fuel, with a default value of 0.00068. ε is the conversion ratio from SO_2_ to sulfate (3.3%). *SN* is the engine exhaust smoke number, obtained from the ICAO emissions database. *AFR* is the air-to-fuel ratio of the engine under different operational phases. *AFR* and δ in different phases during LTO have been listed in Table S2.

### 2.3. Spatial Autocorrelation Analysis Method

In this study, we applied local Moran’s I to reveal the geographical clustering patterns of emissions of air pollutants and CO_2_ from the 10 civil aviation airports. Specifically, Stata 18 (StataCorp LLC, College Station, TX, USA) was used for local Moran’s I analysis. The local Moran’s I for each airport was calculated using Equations (7) and (8).

(7) $$I_{i}=\frac{\left(x_{i}-\overset{¯}{x}\right)}{S^{2}}\sum_{j=1}^{n}W_{ij}\left(x_{j}-\bar{x}\right)$$

(8) $$S^{2}=\frac{1}{n}\sum_{i=1}^{n}(x_{i}-\bar{x}{)}^{2}$$

where $x_{i}$ and $x_{j}$ are the total emissions of the target pollutant for airport i and *j*, $\bar{x}$ is the mean value of all airports, *S*^2^ is the variance, $W_{ij}$ is the spatial weights matrix (using an economic distance matrix in this study), and n is the number of airports in the Shandong airport cluster. The four quadrants of the Moran scatter plot represent four distinct types of local spatial association: points in the first quadrant (H-H) represent spatial units with high values surrounded by neighbors with high values; points in the second quadrant (L-H) indicate units with low values surrounded by neighbors with high values; points in the third quadrant (L-L) denote units with low values surrounded by neighbors with low values; points in the fourth quadrant (H-L) correspond to units with high values surrounded by neighbors with low values.

### 2.4. Multiscale Geographically Weighted Regression Method

In this study, we employed the MGWR 2.2 model (Arizona State University, Tempe, AZ, USA) to investigate the driving factors of spatial heterogeneity of airport aircraft emissions. MGWR model allows each explanatory variable to operate at a distinct spatial bandwidth and provides varying levels of spatial smoothing across different variables, thereby reducing estimation bias and yielding a more realistic and practically useful model of spatial processes. The formula for the MGWR model is as follows:

(9) $$y_{i}=\beta_{0}\left(u_{i},v_{i}\right)+\sum_{j}\beta_{{bw}_{j}}\left(u_{i},v_{i}\right)x_{ij}+\varepsilon_{i}$$

where $y_{i}$ is the emission of air pollutants or CO_2_ at airport i, representing the dependent variable; $x_{ij}$ is independent variable j for airport i; ($u_{i}$, $v_{i}$) corresponds to the geographic coordinates of airport i; ${bw}_{j}$ indicates the bandwidth used for estimating the regression coefficient of the variable *j*, $\beta_{{\mathrm{bw}}_{j}}\left(u_{i},v_{i}\right)$ is the local regression coefficient for the j variable at airport i; $\beta_{0}\left(u_{i},v_{i}\right)$ represents the locally estimated intercept at airport i; $\varepsilon_{i}$ is the error term.

The explanatory variables were selected as potential drivers of spatial variations in airport aircraft emissions, representing regional socioeconomic conditions and airport operational activity. In this study, economic indicators, including Gross Domestic Product (GDP), Consumer Price Index (CPI), and Per Capita Disposable Income (PCDI) of each city, were selected to characterize the socioeconomic and economic conditions of the cities in which the airports are located. These data were collected from the municipal statistical bureaus of Shandong Province. Airport operational indicators, including Air Passenger Throughput (APT), Air Cargo and Mail Throughput (ACMT), and Aircraft Movements (AM) of the 10 civil aviation airports, were selected to represent the intensity of airport aviation activities. These data were obtained from the Statistical Report on Airport Production in East China Civil Airports [24].

In this model, all explanatory variables were first logarithmically transformed to mitigate scale disparities. Then, multicollinearity was assessed using variance inflation factors (VIF) in SPSS Statistics 27 (IBM Corp., Armonk, NY, USA). Table S3 shows that all VIF values remained below 7.5, indicating there was no severe collinearity. During the model calibration process, an adaptive bisquare kernel was adopted as the spatial weighting function. Bandwidth selection was optimized using a golden section search, and initial parameter estimates were derived from a GWR model to enhance the convergence of the multiscale optimization process. Additionally, Python 3.9 (Wilmington, DE, USA) was used for data visualization of the MGWR results.

## 3. Results and Discussion

### 3.1. Total Emissions of Air Pollutants and CO_2_ from Airport Aircraft

The emissions of air pollutants and CO_2_ from aircraft at civil aviation airports in Shandong Province in 2023 have been summarized in Table 1. In this study, NO_x_ emissions are reported as the mass of total NO_x_, rather than as NO_2_ equivalents, consistent with the definition of NO_x_ emission indices in the ICAO EEDB. We found that the total emissions of HC, CO, NO_x_, SO_2_, PM, and CO_2_ were 144.6, 1890.4, 2571.8, 358.7, 18.2, and 584,822 tons, respectively. Notably, the total emissions of NO_x_ were approximately 17.8 times higher than those of HC emissions, primarily due to the distinct combustion conditions associated with different engine operating modes rather than geographical factors. Among the five pollutants, NO_x_ and CO were the dominant pollutants, accounting for 51.6% and 37.9% of total emissions, respectively, while the emission of PM was the lowest one with the proportion of only 0.37%.

At the individual airport level, ZSQD airport exhibited the largest emissions among all airports, and its emissions of HC, CO, NO_x_, SO_2_, and PM were 52.2, 686.4, 960.0, 132.7, and 6.7 tons, respectively, which were similar to the respective values of 55.0, 749.3, 775.1, 67.0, and 5.6 tons estimated by the previous study [17]. Compared with other airports, we found that the emissions from the Shandong airport cluster were significantly lower than those from the Beijing–Tianjin–Hebei airport cluster (Table S4), possibly due to the smaller numbers of airports and flights in Shandong [34]. Moreover, we found that HC, CO, and NO_x_ presented higher emissions in Shandong compared with other large individual airports (e.g., ZHCC, ZBAD, ZGSZ in Table S4) [20,22,35]; while SO_2_ and PM exhibited lower emissions, which might be attributed to the different flight numbers and aircraft fleet composition.

To evaluate the influence of the assumed FSC on PM emission estimates, a sensitivity analysis was conducted by varying the baseline FSC of 0.068% by ±25%. When FSC was decreased and increased by 25%, the estimated total PM emissions decreased from 18.2 to 14.9 tons (−18.1%) and increased to 21.2 tons (+16.5%), respectively (Table S5). This result indicates that FSC has a non-negligible influence on PM emission estimates, mainly through its contribution to the sulfur-related volatile PM component.

**Figure 2:**
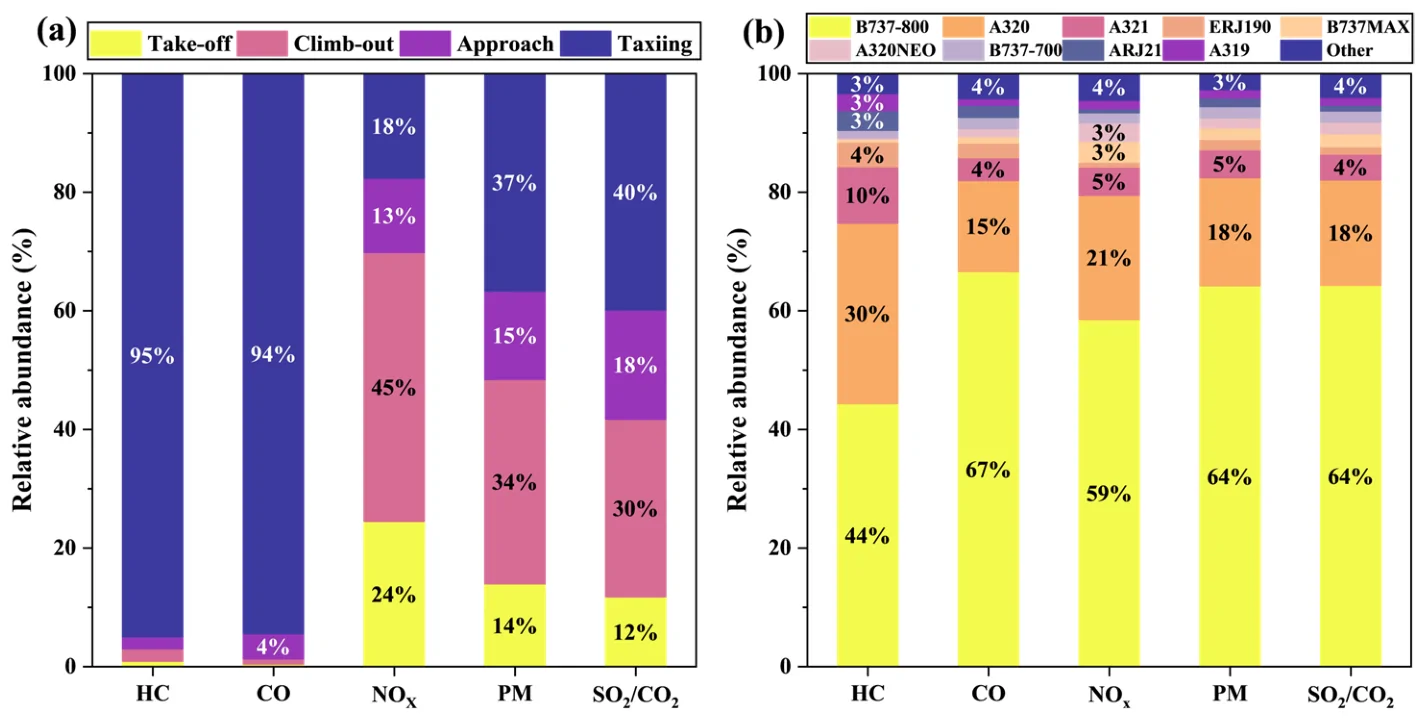
The contributions of different LTO phases (**a**) and different aircraft types (**b**) to the total emissions of five air pollutants and CO_2_ of airport aircraft in Shandong Province.

Our results show that these emission characteristics of aircraft were closely related to the LTO phase and aircraft fleet composition. We found that the taxiing phase was the dominant stage of air pollutant emissions, accounting for 50% of the total emissions of the five pollutants (Figure S3). Figure 2a further shows the contributions of different LTO phases to the total emissions of five air pollutants and CO_2_. We found that CO and HC were mainly emitted from the taxiing phase, which contributed more than 90% of their respective total emissions (Figure 2a). This can be attributed to the low-pressure, low-temperature conditions and the low-thrust setting during the taxiing phase, which promoted incomplete fuel oxidation and thereby significantly increased CO and HC emissions [7]. For NO_x_, nearly 70% of emissions were emitted from the climb-out and take-off phases (Figure 2a), primarily due to the high combustion temperature of aircraft engines operating under high thrust settings, which promotes NO_x_ formation. We found that the contributions of four subphases of the LTO phase to SO_2_ and CO_2_ emissions were the same, because these two pollutants were calculated according to the principle of mass balance and their emissions were directly proportional to fuel consumption. Figure 2a further reveals that SO_2_, CO_2_, and PM were all primarily emitted from taxiing and climb-out phases, and these two phases contributed approximately 70% of their respective total emissions.

Figure 2b shows the contribution of different aircraft types to total emissions of air pollutants and CO_2_. We found that the B737-800 was the largest contributor to five types of air pollutants and CO_2_ emissions, accounting for over 50% of the total emissions of every pollutant except HC (Figure 2b). This result was primarily attributed to the large number of B737-800 aircraft used in the commercial aircraft fleet in Shandong Province (Figure S2). Figure 2b further suggests that the A320 contributed more than 15% of emissions of every pollutant and CO_2,_ and it was the second-largest contributor. Due to the small flight numbers, other aircraft types exhibited relatively small contributions to the emissions. Moreover, we found an interesting result that the total flight sorties of A320 accounted for 75% of those of B737-800 (Figure S2), but the total emissions of CO, NO_x_, SO_2_, PM, and CO_2_ from A320 were only approximately 30% of those from B737-800 (Figure 2b). This divergence between flight sorties and emission levels was primarily attributed to the performance differences in the engines of the A320 and B737-800, exhibiting different emission indices and fuel consumption rates [35,36,37]. Compared with B737-800 only equipped with CFM56-7B, A320 aircraft are generally equipped with various high-bypass-ratio engines, such as the LEAP-1A and PW1000G (Table S6), and these engines exhibit higher combustion efficiencies and lower pollutant emissions [38]. These results also suggest that adjusting and optimizing the aircraft type configuration of the airport fleet will be an effective measure for alleviating aircraft emissions at airports.

### 3.2. Spatiotemporal Heterogeneity of Air Pollutants and CO_2_ Emissions from Airport Aircraft

**Figure 3:**
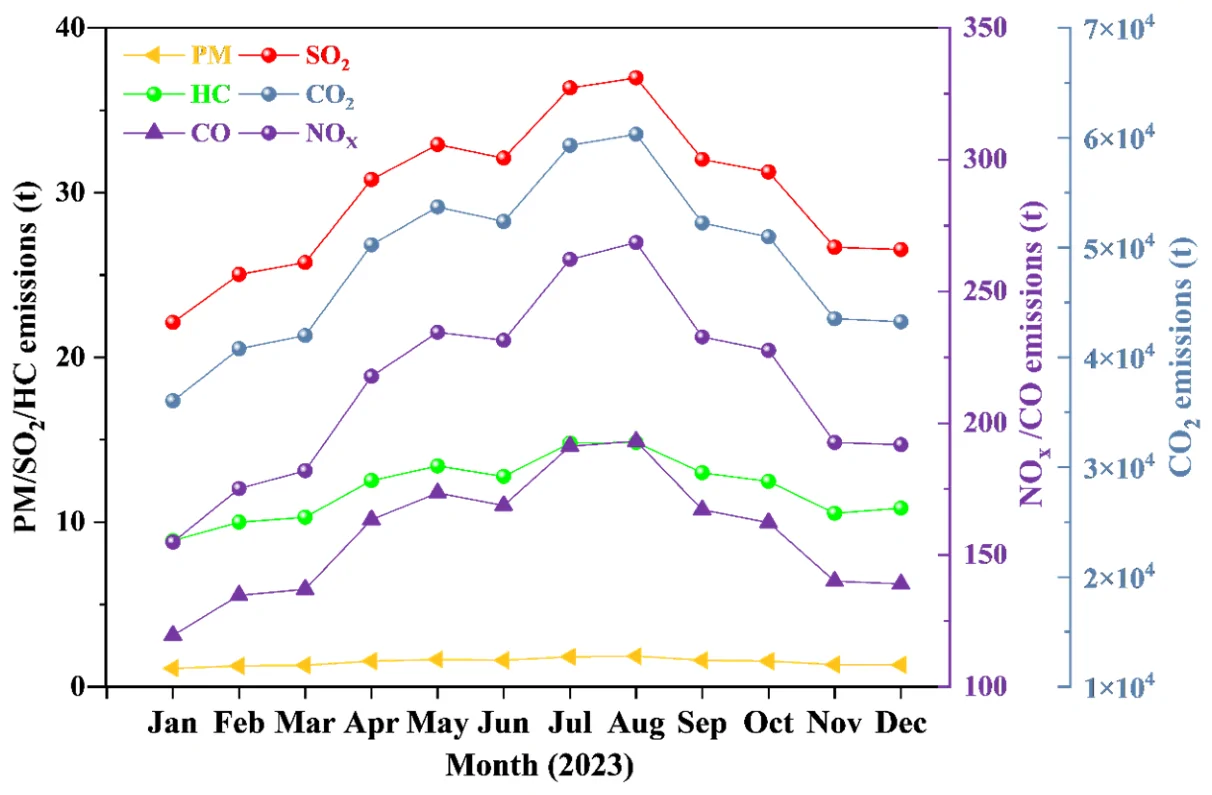
Monthly variation in air pollutants and CO_2_ emissions from airport aircraft in Shandong Province in 2023.

Our results show that the emissions of air pollutants and CO_2_ from airport aircraft exhibited significant temporal variation characteristics. Figure 3 reveals that the emissions of all air pollutants and CO_2_ displayed a prominent upward trend from January onward, reached a distinct peak between July and August, and then presented a gradual downward trend until the end of the year. These results further suggest that the emissions of pollutants and CO_2_ from airport aircraft peak in summer and are at their lowest level in winter. This variation was mainly driven by changes in flight volume. For example, during the summer vacation (July and August), tourism demand could lead to a surge in flight volume to its peak, and consequently, the emissions of air pollutants reach the highest levels (Figure 3 and Figure S4). In addition, the height of the atmospheric boundary layer can also affect these emissions [39]. A higher height of the atmospheric boundary layer in spring and summer (Figure S5) could result in prolonging aircraft climb-out and approach times and then increasing the emissions [20,34,40,41].

**Figure 4:**
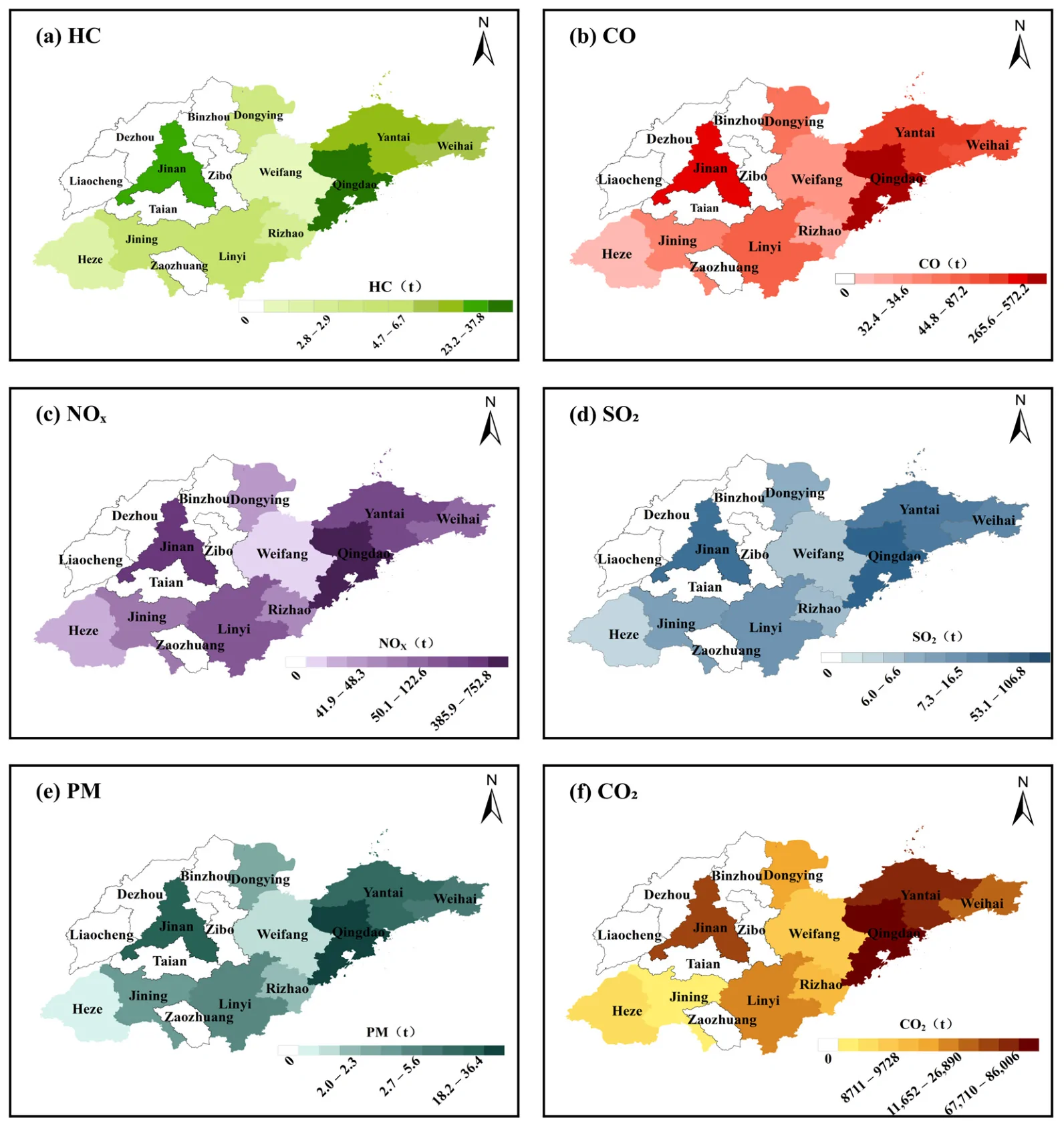
Spatial distribution of air pollutants and CO_2_ emissions from aircraft in civil aviation airports in 10 prefecture-level cities in Shandong Province. There are data gaps in Binzhou, Dezhou, Liaocheng, Taian, Zibo, and Zaozhuang regions due to the absence of civil aviation airports.

Spatial distributions of air pollutants and CO_2_ emissions from airport aircraft in 10 prefecture-level cities in Shandong Province have been illustrated in Figure 4. We found that five types of air pollutants and CO_2_ emissions exhibited similar spatial distribution characteristics, generally characterized by high emissions in the eastern region and low emissions in the western region. Table 1 shows that the emissions of HC, CO, NO_x_, SO_2_, PM, and CO_2_ from airport aircrafts in eastern regions of Shandong Province (including Qingdao Jiaodong International Airport, Yantai Penglai International Airport, and Weihai Dashuipo International Airport) were 84.1, 1049.7, 1468.5, 202.9, 10.3, and 330,757.1 tons, respectively, accounting for 58.2%, 55.5%, 57.1%, 56.6%, 56.4%, and 56.6% of their total emissions, respectively. Among all cities in Shandong Province, Qingdao city exhibited the highest emissions of airport aircraft air pollutants and CO_2_, and Jinan city followed closely, with the second-highest emissions (Figure 4 and Table 1). These two cities jointly contributed more than 66% of the total emissions in Shandong Province (Table 1). This result can be attributed to their large populations and tourist appeal, especially in Qingdao. Moreover, we found that the emissions from the remaining cities were significantly lower than those from these two cities, and Weifang City and Heze City presented the lowest emissions in Shandong Province.

**Figure 5:**
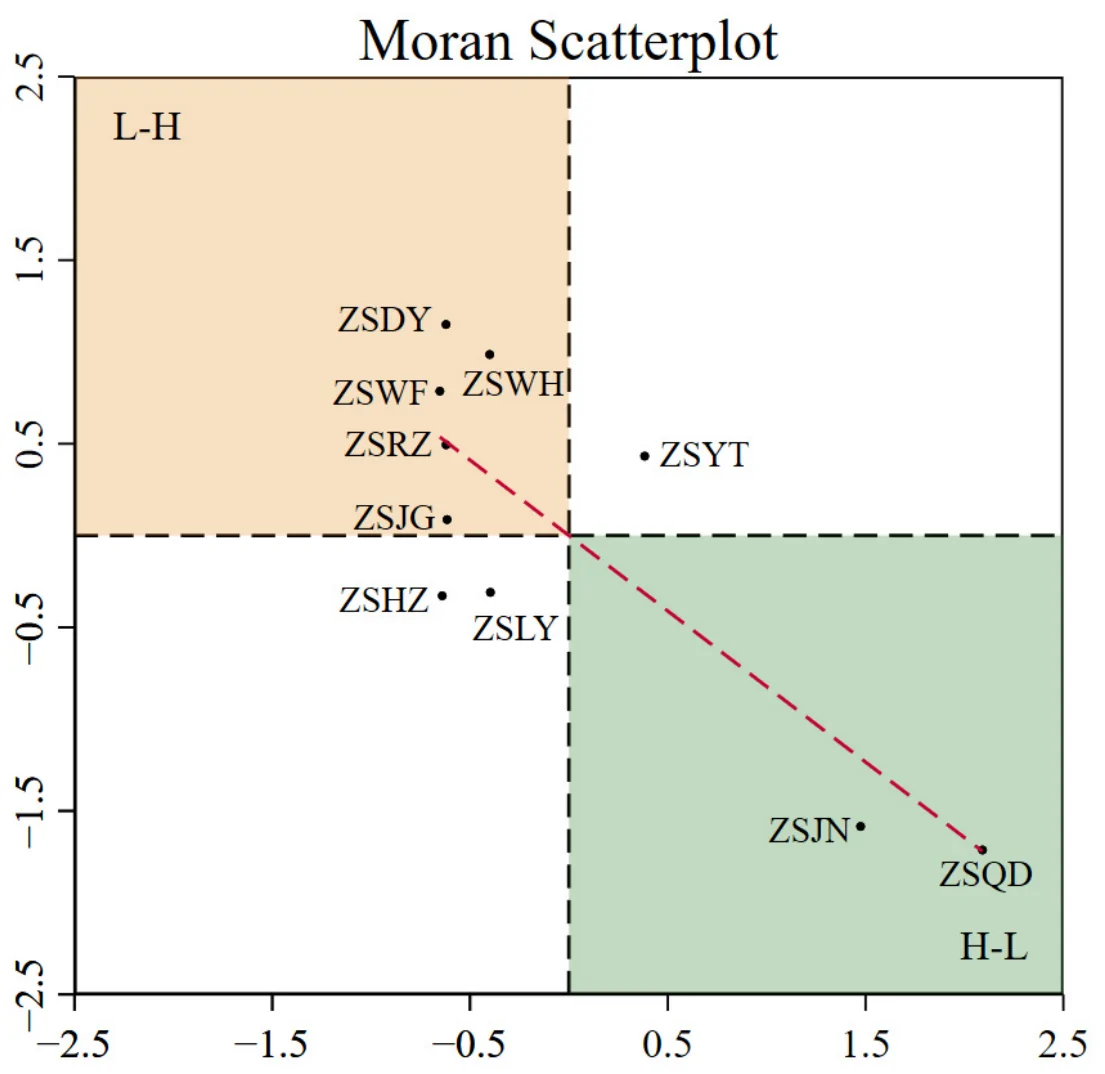
Spatial patterns of local Moran’s I for total aviation emissions from 10 civil aviation airports in Shandong Province in 2023. The x-axis (Z) represents the standardized emission level of an airport, and the y-axis (W_z_) represents the spatially lagged value (i.e., the average emission level of its neighbors). The dashed lines indicate the mean values of Z and W_z_, respectively. The orange and green colors represent L–H and H–L, respectively.

In this study, we also employed a Moran scatterplot to reveal the spatial patterns of these emissions in different airports. Our results show that most airports (including Weihai Dashuipo International Airport, Dongying Shengli Airport, Weifang Nanyuan Airport, Rizhao Shanzihe Airport, Jining Da’an Airport) exhibited distinct “L-H” clustering patterns in the Moran scatterplot (Figure 5), indicating that these airports have relatively low emissions but were surrounded by high-emission hubs. As the two largest airports in Shandong Province, Jinan Yaoqiang International Airport (ZSJN) and Qingdao Jiaodong International Airport (ZSQD) presented “H-L” clustering patterns (Figure 5), suggesting that these airports were surrounded by regions with low emissions. Especially, those cities around Jinan, including Zibo, Taian, Binzhou, Dezhou, and Liaocheng, lacked civil aviation airports with regular commercial flights, leading to their strong reliance on Jinan Yaoqiang International Airport as the central aviation hub (Figure 4). Moreover, Yantai Penglai International Airport (ZSYT) exhibited an “H-H” pattern, while Linyi Qiyang International Airport (ZSLY) and Heze Mudan Airport (ZSHZ) displayed an “L-L” pattern (Figure 5). These spatial patterns reveal distinct disparities in aviation development between the eastern coastal region and the southwestern region in Shandong Province. It should be noted that these clustering results reflect the statistical distribution of emission intensity associated with air traffic activity rather than atmospheric transport or physicochemical processes of emissions. Our results not only reflect the spatial patterns of aircraft emissions in 10 airports, but also reveal the socioeconomic differences between different regions.

### 3.3. Driving Factors of Airport Aircraft Emissions

Our results show that aircraft emissions from 10 civil aviation airports in Shandong Province exhibited obvious temporal and spatial differences. To identify the driving factors behind these variations, the MGWR model was further applied to investigate the underlying relationships between aircraft emissions and socio-economic variables.

The MGWR model produces both global and local regression results, and model performance was assessed using four commonly used goodness-of-fit indicators: R^2^, Adjusted R^2^, corrected Akaike Information Criterion (AICc), and residual sum of squares (RSS). As summarized in Table 2, compared with the global OLS model for all pollutants and CO_2_, we found that the MGWR model has higher R^2^ and Adjusted R^2^, together with lower AICc and RSS values, which indicates that the MGWR model is more capable of capturing the spatial heterogeneity of the relationships between aircraft emissions and socio-economic factors across airports in Shandong Province [42,43]. Therefore, we used MGWR results for further analysis of the driving factors in this study.

**Figure 6:**
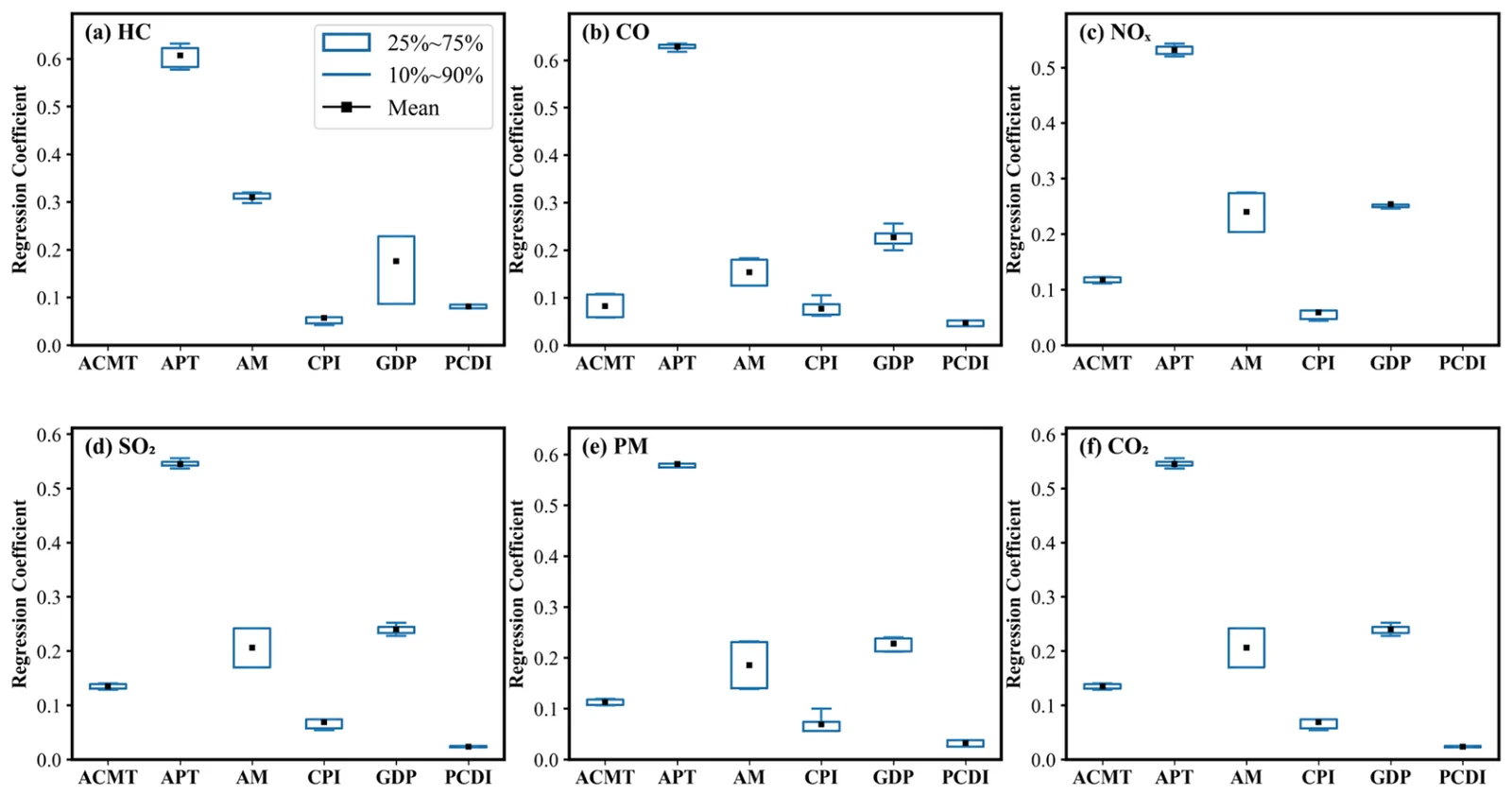
Regression results of the MGWR model for five types of air pollutants and CO_2_. The x-axis is the driving factors (ACMT, APT, AM, CPI, GDP, PCDI), and the y-axis represents regression coefficients. Non-significant data were not presented in the figure, resulting in data gaps therein.

In regression analysis, the mean regression coefficient is commonly used to assess the direction and magnitude of the influence exerted by an independent variable on the dependent variable [44,45]. As shown in Figure 6, APT exhibited the highest regression coefficients among all factors, ranging from 0.532 to 0.629. In terms of statistical significance, APT achieved 100% significance for all air pollutants and CO_2_ (Table S7). These results indicate that APT is the most important driving factor of airport aircraft emissions, and this also means that future growth of air passenger throughput will lead to a continuous increase in aircraft emissions across Shandong Province. Another airport transportation operational factor, AM, also exerted a significant impact on airport aircraft emissions, with the mean regression coefficients ranging from 0.154 to 0.310 (Figure 6). Table S7 shows that AM reached 100% significance for CO, NO_x_, PM, SO_2_, CO_2_, and 80% significance for HC. In contrast, ACMT has the weakest positive effect among the three airports’ transportation operational factors, with mean regression coefficients ranging from 0.082 to 0.135, substantially lower than those of APT and AM (Figure 6).

Among socio-economic factors, GDP emerged as the most impactful economic driver. We found that the GDP factor displayed a statistically significant positive correlation with 100% significance for all pollutants (Table S7), and its mean regression coefficients ranged from 0.176 to 0.254 (Figure 6). These results reveal a strong connection between regional economic development and the transportation sector, and that GDP made a substantial contribution to airport aircraft emissions. In comparison, CPI served as a weak positive driver, and its mean regression coefficients only ranged from 0.057 to 0.077 (Figure 6). Moreover, only 0–30% of PCDI’s coefficients were significant for all air pollutants at the 90% confidence level (Table S7), suggesting its limited influence on airport aircraft emissions. In conclusion, our results indicate that APT, AM, and GDP were the three most important driving factors of airport aircraft emissions.

### 3.4. Atmospheric Implication

This study demonstrates that aviation emissions from Shandong’s airport pose considerable challenges to the regional atmospheric environment. A primary concern is the substantial release of NO_x_, particularly during the climb phase of flights (Table 1 and Figure 2). As a key precursor in photochemical reactions, NO_x_ plays a dominant role in the formation of tropospheric ozone, increasing its concentration and then affecting regional air quality and public health [46,47]. Moreover, although the LTO cycle constitutes the shortest distance in a flight, our analysis reveals that its emissions are disproportionately high. Specifically, CO_2_ emissions from the LTO cycle at only 10 major airports in Shandong Province reached approximately 600,000 tons in 2023 (Table 1), accounting for about 0.6% of the total emissions from Shandong Province’s transportation sector [48]. This notable share highlights the critical importance of implementing targeted mitigation strategies for this specific flight phase.

Here, we propose some targeted emission reduction measures aligned with Shandong’s aviation development characteristics. At the policy level, our study reveals the strong correlation between regional economic development and aviation emissions. Therefore, while vigorously promoting economic development, governments should pay special attention to the environmental impacts of expanding aviation activities. Government departments should formulate relevant policies to balance the relationship between economic growth and aviation emissions, and adopt measures to reduce the increase in aviation emissions driven by economic growth. For example, the European Union incorporated the aviation sector into its Emissions Trading System (EU ETS) in 2012 to reduce regional aviation CO_2_ emissions [49,50].

From the perspective of airports, efforts can be focused on operational refinements, such as optimizing flight schedules and single-engine taxiing to minimize ground congestion and alleviate ground idle time [51]. Moreover, it should be noted that accelerating fleet modernization and adopting sustainable aviation fuel is also a critical pathway for emission reduction. Our results indicate that the B737-800 fleet, widely operated in Shandong Province, has disproportionately high emissions compared to more modern aircraft such as the A320. Notably, sustainable aviation fuel has been demonstrated to exhibit cleaner combustion properties and lower safety risk [52]. Therefore, given that Shandong Province’s narrow-body aircraft fleet is characterized by high utilization rates, replacing obsolete aircraft and upgrading to fuel-efficient aircraft is one of the most effective measures for achieving sustained emission reductions [10].

This study also has several limitations and uncertainties. Firstly, the accuracy of the emission inventory is constrained by its reliance on engine certification database parameters (e.g., emission indices, fuel flow), which may not reflect real-world performance degradation caused by engine aging and wear [7]. Furthermore, the EEDB provides only total HC emission indices without detailed speciation of individual HC species. Therefore, this study quantified only total HC emissions and did not evaluate the environmental impacts of individual HC species. Additionally, using ICAO-standardized TIMs instead of actual operational data can result in non-negligible errors in HC and CO emission estimates, due to the large difference in taxiing durations among airports of different sizes [18]. To reduce this limitation, future studies should incorporate actual operational data to compare with current results, identify the specific discrepancies, and explore the true emission differences.

## 4. Conclusions

In this study, we established a high-resolution emission inventory of air pollutants and CO_2_ for the Shandong Province airport cluster using the ICAO engine emission database and actual flight data. Our results show that the total emissions of HC, CO, NO_x_, SO_2_, PM, and CO_2_ from the airport cluster in Shandong in 2023 were 144.6, 1890.4, 2571.8, 358.7, 18.2, and 584,822 tons, respectively. We found that among the four LTO subphases, the taxiing phase was the dominant contributor to total emissions of air pollutants, especially for HC and CO; and among all aircraft types, the B737-800 emerged as the most common type and the largest emission contributor. Then, the spatiotemporal heterogeneity of air pollutants and CO_2_ emissions from airport aircraft was investigated in this study. Our results reveal that airport aircraft emissions displayed obvious peaks during July and August, but low levels during wintertime. We found that these aviation emissions were highly concentrated in the eastern coastal areas in Shandong Province. Moreover, Qingdao and Jinan exhibited the highest aviation emissions, jointly contributing more than 66% of the total emissions. MGWR model analysis results further demonstrated that this spatiotemporal heterogeneity was largely driven by three key factors: air passenger throughput (APT), aircraft movements (AM), and GDP. Notably, due to the significant similarities in socioeconomic operational patterns and urban development levels across numerous Chinese cities, our findings can be extended to other provinces in China. In the future, policymakers in China should fully incorporate economic conditions, transportation characteristics, and emission intensity to design targeted and differentiated mitigation strategies to effectively reduce aircraft emissions.

## Figures and Tables

**Table 1:** Emissions of aircraft air pollutants and CO_2_ in 10 civil aviation airports in Shandong Province in 2023 (tons).

ICAO Code	Numbers of Flight	HC	CO	NO_x_	SO_2_	PM	CO_2_
ZSQD	156,963	52.2	686.4	960.0	132.7	6.7	216,330.6
ZSJN	128,130	37.8	572.2	752.8	106.8	5.4	174,237.3
ZSYT	64,357	23.2	265.6	385.9	53.1	2.7	86,525.2
ZSWH	22,159	8.7	97.7	122.6	17.1	0.9	27,901.3
ZSLY	20,363	6.7	87.2	123.8	16.5	0.8	26,920.9
ZSJG	9179	4.7	41.1	50.1	7.3	0.4	11,828.9
ZSDY	8902	3.1	44.8	48.3	7.1	0.4	11,652.0
ZSRZ	8069	2.9	32.4	48.5	6.6	0.3	10,724.2
ZSWF	6986	2.5	34.6	37.9	6.0	0.3	9791.5
ZSHZ	6859	2.8	28.4	41.9	5.5	0.3	8910.2
Total	431,967	144.6	1890.4	2571.8	358.7	18.2	584,822.1

**Table 2:** Goodness-of-fit statistics of the OLS and MGWR models for all air pollutants and CO_2_.

Pollutant Type	Model	R^2^	Adjusted R^2^	AICc	RSS
HC	OLS	0.953	0.951	−13.634	5.581
	MGWR	0.986	0.983	−124.587	1.732
CO	OLS	0.977	0.976	−100.639	2.703
	MGWR	0.991	0.989	−176.583	1.115
NO_x_	OLS	0.979	0.978	−109.911	2.502
	MGWR	0.993	0.992	−218.258	0.794
SO_2_	OLS	0.982	0.981	−124.428	2.157
	MGWR	0.993	0.992	−217.936	0.793
PM	OLS	0.981	0.979	−114.729	2.338
	MGWR	0.993	0.991	−206.155	0.875
CO_2_	OLS	0.982	0.981	−124.428	2.157
	MGWR	0.993	0.992	−217.936	0.793

## Data Availability

The data presented in this study are available on request from the corresponding author. The data are not publicly available due to privacy.

## Supplementary Materials

The following supporting information can be downloaded at: https://www.mdpi.com/article/10.3390/toxics14090751/s1, Table S1. Detailed information about 10 major civil aviation airports in Shandong Province; Table S2. Summary of aircraft emission parameters during the LTO cycle; Table S3. Variance inflation factor (VIF), global OLS regression coefficients, and corresponding *p*-values for the six driving factors across different pollutants; Table S4. Comparison of aircraft air pollutants and CO_2_ emissions in different civil aviation airports; Table S5. Sensitivity analysis of PM emissions to variations in FSC across civil aviation airports in Shandong Province; Table S6. Fuel consumption rates of different engines of B737-800 and A320 equipped on mainstream aircraft models; Table S7. Summary of MGWR regression coefficient results for air pollutants and CO_2_; Figure S1. Flight sorties of different aircraft types in 10 civil aviation airports in Shandong Province; Figure S2. Aircraft fleet composition (a) and engine types (b) in 10 civil aviation airports in Shandong Province in 2023; Figure S3. The contributions of four phases of the LTO cycle to total air pollutants emissions of airport aircrafts in Shandong Province; Figure S4. Monthly variation characteristics of arrival, departure, and total flight volumes at airports in Shandong Province in 2023; Figure S5. The height of planetary boundary layer (PBL) of 10 civil aviation airports in Shandong in 2023 (Data source: [39]).

## References

[1] International Air Transport Association COVID-19 Vaccines Must Be Distributed Globally to Save Lives, Protect Jobs. https://www.iata.org/en/pressroom/pressroom-archive/2021-releases/2021-10-04-03/.

[2] International Air Transport Association (2020). IATA Annual Review 2020.

[3] Civil Aviation Administration of China CAAC Statistical Data on Civil Aviation of China 2024. http://www.caacnews.com.cn/1/1/202503/t20250314_1385843.html.

[4] Civil Aviation Administration of China (2023). Civil Aviation Industry Report 2023.

[5] Cui Q., Lei Y., Li Y., Wanke P.F. (2022). Impacts of the COVID-19 on all aircraft emissions of international routes in South America. iScience.

[6] Airbus Global Market Forecast 2023–2042. https://www.noticiaslatamsales.com/noticias/november-2023/airbus-global-market-forecast/.

[7] Aygun H., Turan O. (2023). Analysis of cruise conditions on energy, exergy and NOx emission parameters of a turbofan engine for middle-range aircraft. Energy.

[8] Lyu C., Liu X., Wang Z., Yang L., Liu H., Yang N., Xu S., Cao L., Zhang Z., Pang L. (2023). An emissions inventory using flight information reveals the long-term changes of aviation CO_2_ emissions in China. Energy.

[9] Han Y., Shen H., He X., Mai Z., Zhang R., Zheng Z., Liu Y., Zhang X., Li G., Zhang Z. (2025). A Comprehensive Analysis of Interflight Variability in Carbon Dioxide Emissions from Global Aviation. Environ. Sci. Technol..

[10] Zhang J., Zhang J., Yan J., Nielsen C.P., Wu Y., Hao J., Zhang S., McElroy M.B. (2025). Rising CO_2_ and Decarbonization Strategies of Aviation in China in the Coming Decade. Environ. Sci. Technol..

[11] Zhang J., Zhang S., Zhang X., Wang J., Wu Y., Hao J. (2022). Developing a High-Resolution Emission Inventory of China’s Aviation Sector Using Real-World Flight Trajectory Data. Environ. Sci. Technol..

[12] Hudda N., Durant L.W., Fruin S.A., Durant J.L. (2020). Impacts of Aviation Emissions on Near-Airport Residential Air Quality. Environ. Sci. Technol..

[13] Moore R.H., Shook M.A., Ziemba L.D., DiGangi J.P., Winstead E.L., Rauch B., Jurkat T., Thornhill K.L., Crosbie E.C., Robinson C. (2017). Take-off engine particle emission indices for in-service aircraft at Los Angeles International Airport. Sci. Data.

[14] Zhang X., Karl M., Zhang L., Wang J. (2020). Influence of Aviation Emission on the Particle Number Concentration near Zurich Airport. Environ. Sci. Technol..

[15] Hu S., Fruin S., Kozawa K., Mara S., Winer A.M., Paulson S.E. (2009). Aircraft Emission Impacts in a Neighborhood Adjacent to a General Aviation Airport in Southern California. Environ. Sci. Technol..

[16] Yim S.H.L., Lee G.L., Lee I.H., Allroggen F., Ashok A., Caiazzo F., Eastham S.D., Malina R., Barrett S.R.H. (2015). Global, regional and local health impacts of civil aviation emissions. Environ. Res. Lett..

[17] Liu H., Tian H., Hao Y., Liu S., Liu X., Zhu C., Wu Y., Liu W., Bai X., Wu B. (2019). Atmospheric emission inventory of multiple pollutants from civil aviation in China: Temporal trend, spatial distribution characteristics and emission features analysis. Sci. Total Environ..

[18] Lu B., Dong J., Wang C., Sun H., Yao H. (2024). High-resolution spatio-temporal estimation of CO_2_ emissions from China’s civil aviation industry. Appl. Energy.

[19] Ma S., Wang X., Han B., Zhao J., Guan Z., Wang J., Zhang Y., Liu B., Yu J., Feng Y. (2024). Exploring emission spatiotemporal pattern and potential reduction capacity in China’s aviation sector: Flight trajectory optimization perspective. Sci. Total Environ..

[20] Han B., Wang L., Deng Z., Shi Y., Yu J. (2022). Source emission and attribution of a large airport in Central China. Sci. Total Environ..

[21] Moradi H., Shafabakhsh G., Naderan A. (2024). Effect of airport pollution on airport cities and air quality of the area (case study: Imam Khomeini international airport). J. Transp. Health.

[22] Lin C., Han Y., Cao C., Wang Y., Zhang R., He X., Li G., Zhang Z., Liang Z., Zhou Z. (2025). Assessing the Impacts of Aircraft Emissions on Air Quality in a Megacity Using High-Resolution Flight Tracking Data. J. Geophys. Res. Atmos..

[23] Shandong Provincial Government Transport and General Aviation Advance in Parallel: Shandong Airports “Two Wings” Take Off. http://www.shandong.gov.cn/art/2024/12/7/art_97564_659235.html.

[24] Civil Aviation Administration of China (2024). 2023 Business Volume Statistics of East China Civil Aviation Airports (Provincial Ranking).

[25] Ma S.M., Zheng W.H., Han B., Zhao J.B., Yu J. (2025). Emission characteristics and environmental impact assessment of a large airport in eastern coastal area. China Environ. Sci..

[26] Aygun H., Caliskan H. (2021). Environmental and enviroeconomic analyses of two different turbofan engine families considering landing and take-off (LTO) cycle and global warming potential (GWP) approach. Energy Convers. Manag..

[27] Wang H., Sheng N., Song Q., Xu L., Bai J. (2025). Exploring the potential carbon emissions and net-zero path of international flights: A case study in Macao. J. Environ. Sci..

[28] Zhang X., Chen X., Wang J. (2019). A number-based inventory of size-resolved black carbon particle emissions by global civil aviation. Nat. Commun..

[29] International Civil Aviation Organization ICAO Aircraft Engine Emissions Databank. https://www.easa.europa.eu/en/domains/environment/icao-aircraft-engine-emissions-databank.

[30] An H., Wang Y., Wang Y., Liu J., Tang X., Yi H. (2025). Civil aviation emissions in China in 2019: Characteristics and abatement potential. J. Environ. Sci..

[31] Wen C., Lang J., Fu Y., Yang Z., Cheng X., Zhou Y., Zhang S., Chen D., Cheng S. (2025). Refined aircraft landing-takeoff activity modeling to improve the estimation of aviation CO_2_ and pollutants emissions. npj Clim. Atmos. Sci..

[32] Stettler M.E.J., Eastham S., Barrett S.R.H. (2011). Air quality and public health impacts of UK airports. Part I: Emissions. Atmos. Environ..

[33] Wayson R.L., Fleming G.G., Iovinelli R. (2009). Methodology to Estimate Particulate Matter Emissions from Certified Commercial Aircraft Engines. J. Air Waste Manag. Assoc..

[34] Han B., Kong W.K., Yao T.W., Wang Y. (2020). Air Pollutant Emission Inventory from LTO Cycles of Aircraft in the Beijing-Tianjin-Hebei Airport Group, China. Environ. Sci..

[35] Wang Y.-n., Zou C., Fang T.-g., Sun N.-x., Liang X.-y., Wu L., Mao H.-j. (2023). Emissions from international airport and its impact on air quality: A case study of beijing daxing international airport (PKX), China. Environ. Pollut..

[36] Ekici S., Sevinc H. (2022). Understanding a commercial airline company: A case study on emissions and air quality costs. Int. J. Environ. Sci. Technol..

[37] Maes J., Bezantakos S., Kavabata L.K., Biskos G., Dedoussi I.C. (2025). Aircraft emissions of ultrafine particles characterized by real-world near runway measurements. npj Clim. Atmos. Sci..

[38] Zhao Y., Li G., Zhou L., Zhang Z., Gao G., Chang L., Chen L. (2026). Comparative study of aviation nvPM emissions using quick access recorder data. Transp. Res. Part D Transp. Environ..

[39] Hersbach H.B.B., Berrisford B., Biavati G., Horányi A., Muñoz Sabater J., Nicolas J., Peubey C., Radu R., Rozum I., Schepers D. (2023). ERA5 Hourly Data on Single Levels from 1940 to Present. https://cds.climate.copernicus.eu/datasets/reanalysis-era5-single-levels?tab=overview.

[40] Han B., He Z., Zhang D., Kong W.K., Wang Y. (2020). Research on aircraft LTO pollutant emission factors and emission inventory in Guangdong-Hong Kong-Macao Greater Bay Area, China. China Environ. Sci..

[41] Zhou Y., Jiao Y., Lang J., Chen D., Huang C., Wei P., Li S., Cheng S. (2019). Improved estimation of air pollutant emissions from landing and takeoff cycles of civil aircraft in China. Environ. Pollut..

[42] He C., Niu X., Ye Z., Wu Q., Liu L., Zhao Y., Ni J., Li B., Jin J. (2023). Black carbon pollution in China from 2001 to 2019: Patterns, trends, and drivers. Environ. Pollut..

[43] Zhao P., Li Z., Xiao Z., Jiang S., He Z., Zhang M. (2023). Spatiotemporal characteristics and driving factors of CO_2_ emissions from road freight transportation. Transp. Res. Part D Transp. Environ..

[44] Feng L., Yang W., Hu J., Wu K., Li H. (2025). Exploring the nexus between rural economic digitalization and agricultural carbon emissions: A multi-scale analysis across 1607 counties in China. J. Environ. Manag..

[45] Shi X., Ling G.H.T., Leng P.C., Rusli N., Matusin A.M.R.A. (2023). Associations between institutional-social-ecological factors and COVID-19 case-fatality: Evidence from 134 countries using multiscale geographically weighted regression (MGWR). One Health.

[46] Elshorbany Y., Ziemke J.R., Strode S., Petetin H., Miyazaki K., De Smedt I., Pickering K., Seguel R.J., Worden H., Emmerichs T. (2024). Tropospheric ozone precursors: Global and regional distributions, trends, and variability. Atmos. Chem. Phys..

[47] Fry M.M., Naik V., West J.J., Schwarzkopf M.D., Fiore A.M., Collins W.J., Dentener F.J., Shindell D.T., Atherton C., Bergmann D. (2012). The influence of ozone precursor emissions from four world regions on tropospheric composition and radiative climate forcing. J. Geophys. Res. Atmos..

[48] Xu R., Tong D., Xiao Q., Qin X., Chen C., Yan L., Cheng J., Cui C., Hu H., Liu W. (2024). MEIC-global-CO_2_: A new global CO_2_ emission inventory with highly-resolved source category and sub-country information. Sci. China Earth Sci..

[49] Pietzcker R.C., Osorio S., Rodrigues R. (2021). Tightening EU ETS targets in line with the European Green Deal: Impacts on the decarbonization of the EU power sector. Appl. Energy.

[50] Zhang Y., Wang K. (2024). Mitigation effect of the European Union emission trading system on aviation emissions. Transp. Res. Part D Transp. Environ..

[51] Xu Y., Kang M., Chen D., Wu C., Wang X., Tu R., Yu W. (2025). Flight departure optimization reduces airport CO_2_ emissions proved by Beijing-Tianjin-Hebei multiple-airport-region. Transp. Res. Part D Transp. Environ..

[52] Durdina L., Decker Z.C.J., Edebeli J., Spirig C., Frischknecht T., Anet J.G., Brem B.T., Siegerist F., Rindlisbacher T. (2025). Gaseous and Particulate Emissions from a Small Business Jet Using Conventional Jet A-1 and a 30% SAF Blend. ACS ES&T Air.

