# Supplementary material for: Spatiotemporal Emission Characteristics and Driving Factors of Air Pollutants and CO2 from Aircraft at Airports: A Case Study in China
Source: Toxics. 2026 Aug 26;14(9):751. doi: 10.3390/toxics14090751 (PMC13611485; doi:10.3390/toxics14090751)
Supplement: Supplementary file 1 [file toxics-14-00751-s001.zip › toxics-4497479-supplementary.pdf]

Supporting Materials

Spatiotemporal emission characteristics and driving factors of airport aircraft air pollutants and CO_2: a case study in China

Yapeng Li[1], Mingwei Zhang[1], Yinxiao Zhang[1,2*], Zhongcun Han[3], Jian Chen[1], Yuzhe Liu[1], Runjie Liu[1], Jiahui Xu[1], Yueyuan Niu[1], Wei Hao[1], Wenlai Ma[1], Weijun Li[4]

[1]Flight College, Shandong University of Aeronautics, Binzhou, 256600, China

[2]Shandong Key Laboratory of Eco-Environmental Science for the Yellow River Delta, Shandong University of Aeronautics, Binzhou, 256600, China

[3]CAST Zhongyu (Beijing) New Technology Development Co. Ltd, Beijing, 101200, China

[4]Department of Atmospheric Sciences, School of Earth Sciences, Zhejiang University, Hangzhou, 310027, China

*Corresponding Email: zhangyinxiao@sdua.edu.cn

Table S1. Detailed information about 10 major civil aviation airports in Shandong Province.

IATA	ICAO	Airport Name	Geographic Coordinates	City	Airfield Area Class
TAO	ZSQD	Qingdao Jiaodong International Airport	36°21′55″N, 120°05′43″E	Qingdao	4F
TNA	ZSJN	Jinan Yaoqiang International Airport	36°51′07″N, 117°12′49″E	Jinan	4E
YNT	ZSYT	Yantai Penglai International Airport	37°39′36″N, 120°59′28″E	Yantai	4E
WEH	ZSWH	Weihai Dashuipo International Airport	37°11′20″N, 122°14′22″E	Weihai	4D
LYI	ZSLY	Linyi Qiyang International Airport	35°02′55″N, 118°24′55″E	Linyi	4D
JNG	ZSJG	Jining Da’an Airport	35°17′52″N, 116°21′23″E	Jining	4C
RIZ	ZSRZ	Rizhao Shanzihe Airport	37°30′21″N, 118°47′15″E	Rizhao	4C
WEF	ZSWF	Weifang Nanyuan Airport	35°24′06″N, 119°19′45″E	Weifang	4D
DOY	ZSDY	Dongying Shengli Airport	36°38′31″N, 119°07′12″E	Dongying	4D
HZA	ZSHZ	Heze Mudan Airport	35°12′50″N, 115°44′17″E	Heze	4C

Table S2. Summary of aircraft emission parameters during the LTO cycle.

Mode	Thrust power setting	TIM（min）	AFR	δ (mg/g)
Take-off	100%	0.7	45	115
Climb-out	85%	2.2	51	76
Approach	30%	4	83	56.25
Taxiing	7%	26	106	6.17

Table S3. Variance inflation factor (VIF), global OLS regression coefficients, and corresponding p-values for the six driving factors across different pollutants.

Pollutant Type		ACMT	APT	AM	CPI	GDP	PCDI
	VIF	4.025	7.405	2.612	1.605	3.521	1.412
HC	Coefficients	0.092	0.797	0.046	0.02	0.087	0.011
	P-value	0.024	0	0.157	0.038	0.023	0.636
CO	Coefficients	0.171	0.655	0.124	0.04	0.106	0.047
	P-value	0	0	0	0.006	0	0.005
NO_x	Coefficients	0.185	0.628	0.183	0.03	0.108	-0.004
	P-value	0	0	0	0.033	0	0.816
SO_2	Coefficients	0.19	0.619	0.167	0.037	0.118	0.015
	P-value	0	0	0	0.004	0	0.314
PM	Coefficients	0.18	0.653	0.145	0.036	0.096	0.025
	P-value	0	0	0	0.009	0	0.103
CO_2	Coefficients	0.199	0.61	0.167	0.037	0.118	0.015
	P-value	0	0	0	0.004	0	0.314

Table S4. Comparison of aircraft air pollutants and CO_2 emissions in different civil aviation airports.

Airport	Year	HC	CO	NO_x	SO_2	PM	CO_2	Reference
ZSQD	2015	55.0	749.3	775.1	67.0	5.6	—	[17]
Beijing-Tianjin-Hebei Airport Cluster	2018 – 2019	508.0	3,972.2	10,720.5	407.8	53.7	—	[34]
ZHCC	2019	56.3	577.1	969.8	268.3	26.9	—	[20]
ZBAD	2020 – 2021	138.0	1150.0	1,760.0	116.0	35.3	375,000	[35]
ZGSZ	2021	98.7	1,281.3	3,080.5	156.6	17.0	—	[22]
Shandong Province Airport Cluster	2023	144.4	1,890.4	2,571.8	121.9	18.1	584,822.2	This study

Note: The full name of the individual airport of ZHCC, ZSAD and ZGSZ is Zhengzhou Xinzheng, Beijing Daxing, Guangzhou Baiyun international airports, respectively.

Table S5. Sensitivity analysis of PM emissions to variations in FSC across civil aviation airports in Shandong Province.

Scenario	FSC	Total PM (t)	Change relative to baseline (%)
Low	0.051%	14.9	−18.1
Baseline	0.068%	18.2	—
High	0.085%	21.2	16.5

Note: The baseline FSC was 0.068%. The low and high FSC scenarios correspond to 25% decreases and increases from the baseline value, respectively. All other parameters were kept unchanged.

Table S6. Fuel consumption rates of different engines of B737-800 and A320 equipped on mainstream aircraft models.

Aircraft type	Engine type	Fuel flow (kg/s)			
		Take off	Climb out	Approach	Taxiing
B737-800	CFM56-7B	1.293	1.031	0.343	0.11
A320	CFM56-5B	1.166	0.961	0.326	0.107
	LEAP-1A	1.062	0.869	0.284	0.098
	PW1000G	0.71	0.6	0.21	0.08
	PW1100G	0.88	0.73	0.2546	0.08
	PW1127G	0.88	0.73	0.2546	0.08
	V2500	1.113	0.924	0.334	0.124

Table S7. Summary of MGWR regression coefficient results for air pollutants and CO_2.

Pollutant Type	Statistical Indicator	Driver factors					
		ACMT	APT	AM	CPI	GDP	PCDI
HC	Mean	0.038	0.587	0.249	0.034	0.183	0.017
	Min	0.027	0.393	0.01	-0.024	0.168	-0.008
	Max	0.053	0.655	0.328	0.089	0.22	0.085
	% sig.	0%	100%	80%	50%	100%	20%
CO	Mean	0.074	0.599	0.151	0.048	0.231	0.022
	Min	0.058	0.426	0.107	-0.004	0.16	0.005
	Max	0.108	0.635	0.183	0.105	0.289	0.052
	% sig.	100%	100%	100%	50%	100%	30%
NO_x	Mean	0.117	0.507	0.242	0.037	0.249	-0.003
	Min	0.111	0.358	0.204	0.012	0.175	-0.006
	Max	0.123	0.543	0.275	0.091	0.289	0.003
	% sig.	100%	100%	100%	50%	100%	0%
SO_2	Mean	0.135	0.52	0.211	0.044	0.236	0.01
	Min	0.129	0.366	0.17	0.009	0.17	-0.002
	Max	0.14	0.556	0.242	0.102	0.273	0.025
	% sig.	100%	100%	100%	50%	100%	20%
PM	Mean	0.113	0.554	0.194	0.044	0.224	0.014
	Min	0.106	0.39	0.139	0.005	0.154	0
	Max	0.119	0.593	0.232	0.1	0.266	0.038
	% sig.	100%	100%	100%	50%	100%	30%
CO_2	Mean	0.135	0.52	0.211	0.044	0.236	0.01
	Min	0.129	0.366	0.17	0.009	0.17	-0.002
	Max	0.14	0.556	0.242	0.102	0.273	0.025
	% sig.	100%	100%	100%	50%	100%	20%

* % sig. representing the proportion of significant cases at the 90% confidence level.

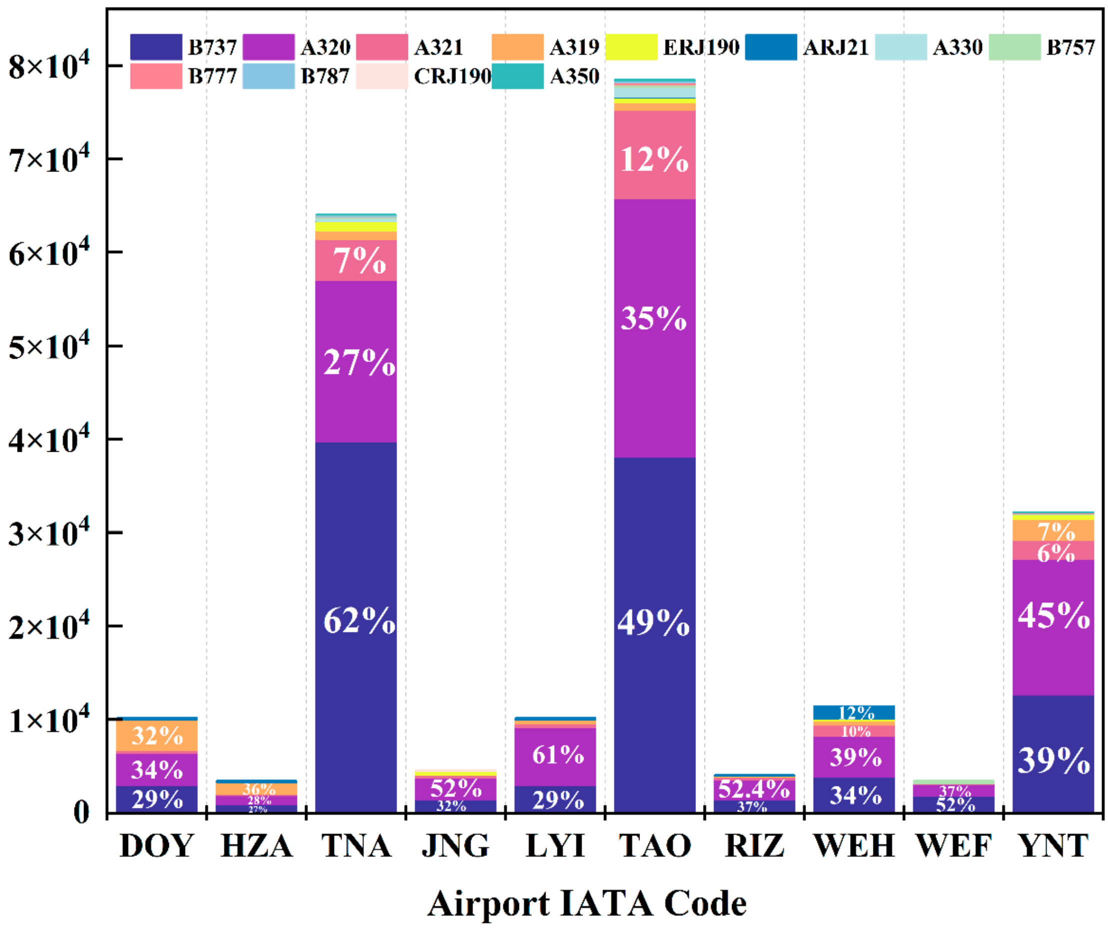

Figure S1. Flight sorties of different aircraft types in 10 civil aviation airports in Shandong Province.

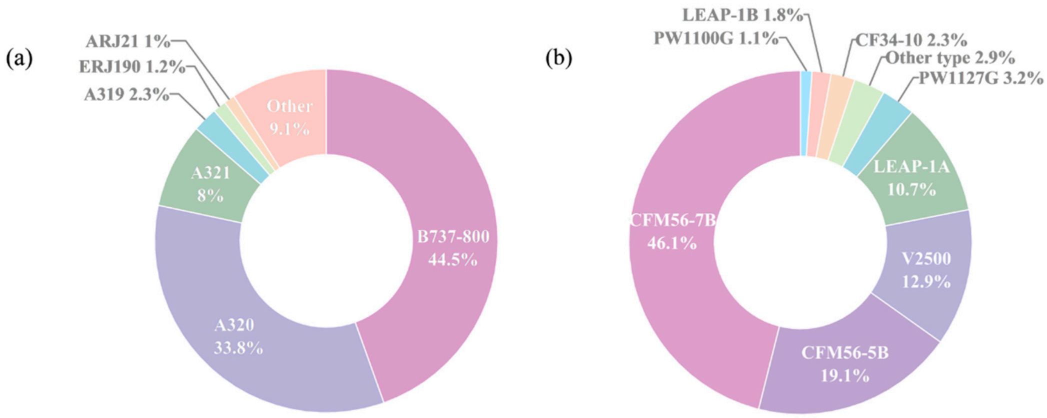

Figure S2. Aircraft fleet composition (a) and engine types (b) in 10 civil aviation airports in Shandong Province in 2023.

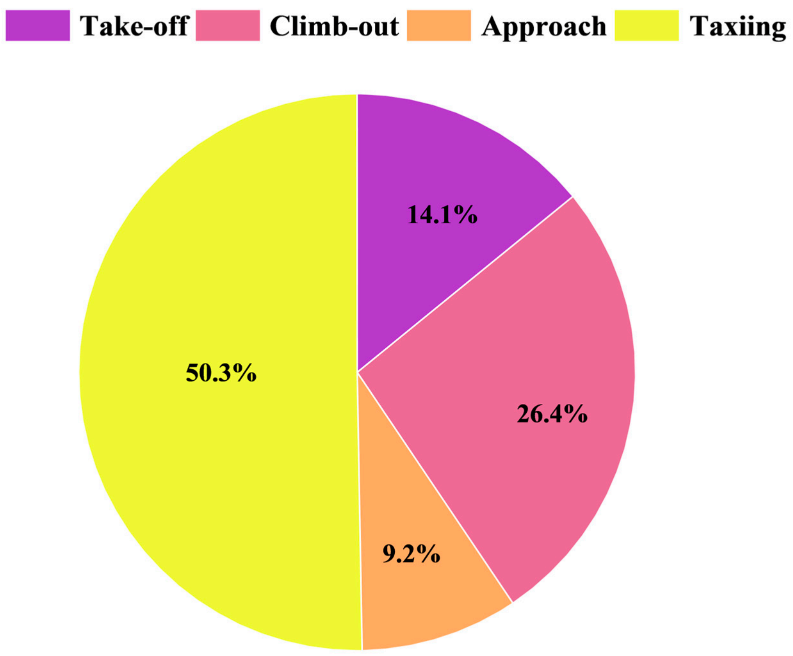

Figure S3. The contributions of four phases of the LTO cycle to total air pollutants emissions of airport aircrafts in Shandong Province.

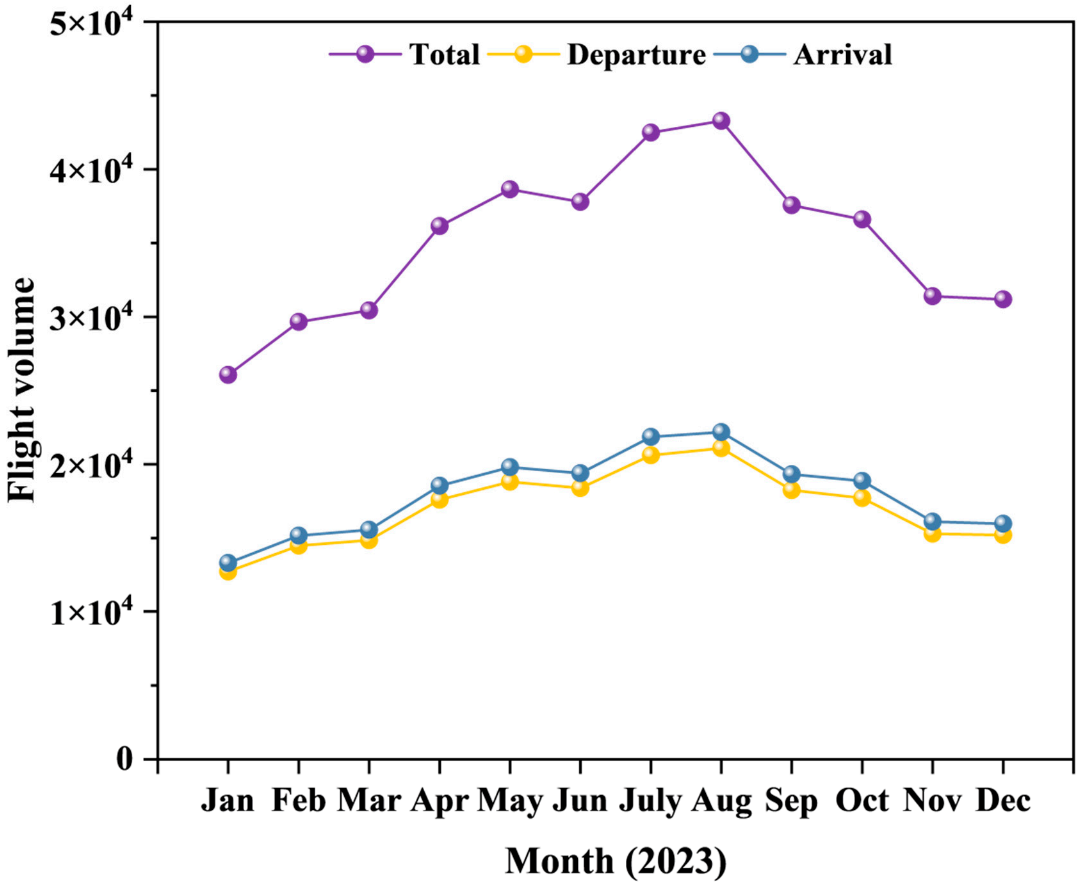

Figure S4. Monthly variation characteristics of arrival, departure, and total flight volumes at airports in Shandong Province in 2023.

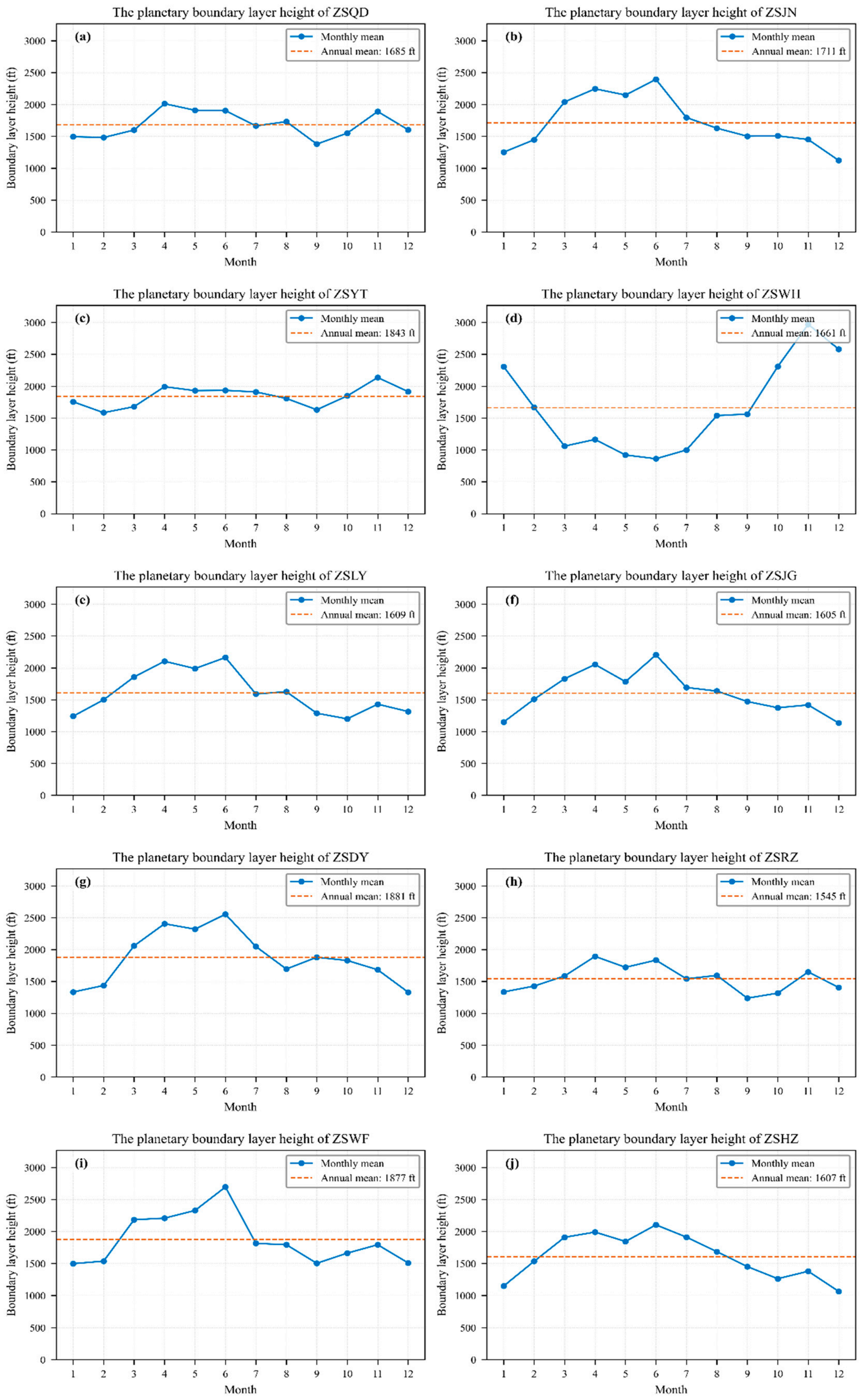

Figure S5. The height of planetary boundary layer (PBL) of 10 civil aviation airports in Shandong in 2023 (Data source: [39]).
